# Everolimus-induced epithelial to mesenchymal transition in immortalized human renal proximal tubular epithelial cells: key role of heparanase

**DOI:** 10.1186/1479-5876-11-292

**Published:** 2013-11-20

**Authors:** Valentina Masola, Gianluigi Zaza, Simona Granata, Giovanni Gambaro, Maurizio Onisto, Antonio Lupo

**Affiliations:** 1 Renal Unit, Department of Medicine, University-Hospital of Verona, Piazzale A. Stefani 1, 37126 Verona, Italy; 2 Division of Nephrology and Dialysis, Columbus-Gemelli University Hospital, Renal Program, Catholic University, Via Moscati 31, 00168 Roma, Italy; 3 Department of Biomedical Sciences, University of Padova, Via Colombo 3, 35121 Padova, Italy

**Keywords:** Epithelial-mesenchymal transition, Everolimus, Heparanase, mTOR, Tubular cells

## Abstract

**Background:**

Everolimus (EVE) is a drug widely used in several renal transplant protocols. Although characterized by a relatively low nephrotoxicity, it may induce several adverse effects including severe fibro-interstitial pneumonitis. The exact molecular/biological mechanism associated to these pro-fibrotic effects is unknown, but epithelial to mesenchymal transition (EMT) may have a central role. Additionally, heparanase, an enzyme recently associated with the progression of chronic allograft nephropathy, could contribute to activate this machinery in renal cells.

**Methods:**

Several biomolecular strategies (RT-PCR, immunofluorescence, zymography and migration assay) have been used to assess the capability of EVE (10, 100, 200 and 500 nM) to induce an *in vitro* heparanase-mediated EMT in wild-type (WT) and Heparanase (HPSE)-silenced immortalized human renal epithelial proximal tubular cells (HK-2). Additionally, microarray technology was used to find additional biological elements involved in EVE-induced EMT.

**Results:**

Biomolecular experiments demonstrated a significant up-regulation (more than 1.5 fold increase) of several genes encoding for well known EMT markers [(alpha-smooth muscle actin (α-SMA), Vimentin (VIM), Fibronectin (FN) and matrix metalloproteinase-9 (MMP9)], enhancement of MMP9 protein level and increment of cells motility in WT HK2 cells treated with high concentrations of EVE (higher than 100 nM). Similarly, immunofluorescence analysis showed that 100 nM of EVE increased α-SMA, VIM and FN protein expression in WT HK2 cells. All these effects were absent in both HPSE- and AKT-silenced cell lines. AKT is a protein having a central role in EMT. Additionally, microarray analysis identified other 2 genes significantly up-regulated in 100 nM EVE-treated cells (p < 0.005 and FDR < 5%): transforming growth factor beta-2 (TGFβ2) and epidermal growth factor receptor (EGFR). Real-time PCR analysis validated microarray.

**Conclusions:**

Our *in vitro* study reveals new biological/cellular aspects of the pro-fibrotic activity of EVE and it demonstrates, for the first time, that an heparanase-mediated EMT of renal tubular cells may be activated by high doses of this drug. Additionally, our results suggest that clinicians should administer the adequate dosage of EVE in order to increase efficacy and reduce adverse effects. Finally heparanase could be a new potential therapeutic target useful to prevent/minimize drug-related systemic fibrotic adverse effects.

## Introduction

Everolimus (EVE) belongs to the group of drugs called mammalian target of rapamycin inhibitors (mTOR-I), a group of proliferation signal inhibitors used in several *de novo* and maintenance renal transplant immunosuppressive protocols and to treat some tumors [1].

The main mechanism of action of this drug is the inhibition of mTOR, a regulatory protein kinase involved in lymphocyte proliferation, developmental processes such as neurologic and muscle generation, and tumor cell growth [2,3]. The anticancer efficacy is also correlated to the up-regulation of adhesion molecules, a switch to less invasive phenotype of tumoral cells and the inhibition of angiogenesis is due to the reduction of vascular endothelial growth factor production and the decrease of endothelial sensitivity to such growth factor [4-6]. Additionally, antineoplastic properties are enhanced by the inhibition of the crosstalk among mTORC1, mTORC2 and Phosphatidylinositol-3 kinase (PI3K) [7-9].

Moreover, because of its relative low nephrotoxicity, EVE is a valid option to calcineurin inhibitors for maintenance immune suppression in patients with chronic allograft nephropathy [10].

Although it is clear the clinical utility of this drug category, as other antineoplastic/immunosuppressive drugs, mTOR-I may induce the development of several renal (e.g., proteinuria) and systemic side effects including hematological disorders (e.g., anemia, leukopenia and thrombocytopenia), dismetabolism (e.g., hyperlipidemia, post-transplant diabetes), lymphedema, stomatitis and fertility/gonadic toxicity [11-13].

In the last years, numerous reports have shown fibrosis-related pulmonary adverse effects (e.g., lymphocytic interstitial pneumonitis, bronchiolitis obliterans with organizing pneumonia and focal pulmonary fibrosis) in oncological and renal transplant patients treated with mTOR-I [13-17]. It is well known that in this clinical condition, epithelial to mesenchymal transition (EMT) have a pivotal role [18-20].

The EMT is a phenotypic conversion of epithelium to a fibroblastic or myofibroblastic phenotype. Cells loose their epithelial proteins and acquire new mesenchymal markers [including alpha-smooth muscle actin (α-SMA), vimentin (VIM) and fibronectin (FN)], decrease intercellular adhesion, modify cell polarity and, finally, increase migratory and invasive properties [21].

Moreover, in renal tissue, during EMT, tubular cells acquire the capacity to migrate into the interstitium through the degradation of basement membrane [22,23]. This event is sustained by the release of matrix metalloproteinases (MMPs) [24,25] and heparanase (HPSE) [26-28], an endoglycosidase that cleaves heparan sulphate chains involved in the pathogenesis of several proteinuric nephropathies [29,30] and onset of chronic allograft dysfunction [31].

Although EMT program is not the only biological mechanism involved in the myofibroblast genesis in renal tissue [32,33], it could represent a substantial portion of the pro-fibrotic machinery induced by EVE.

Therefore, the aim of our study has been to analyze whether EVE (10, 100, 200 and 500 nM) was able to induce *in vitro* EMT in immortalized human tubular epithelial cells (HK2) and to assess the relative contribution of HPSE to this biological effect. Additionally, it could be useful to better understand the complex cellular machinery associated with the onset of renal or systemic fibrosis-related adverse effects following the administration of this drug.

## Material and methods

### Cell cultures, HPSE and AKT silencing and treatments

Everolimus was kindly provided by Novartis (Basel, Switzerland) and dissolved in DMSO according to the manufacturer’s instructions. Clonal human derived renal proximal tubule (HK-2) cells were grown in DMEM-F12 (EuroClone) (17.5 mM glucose) supplemented with 10% fetal bovine serum (Sigma Aldrich), 2 mM L-glutamine, penicillin (100 U/ml) and streptomycin (100 μg/ml) and maintained at 37°C in a 5% CO_2_ water-saturated atmosphere.

A stably HPSE-silenced HK-2 cell line was obtained by transfection with shRNA plasmid targeting human HPSE (NM_006665) purchased from OriGene, as previously described [27]. HPSE-silenced HK-2 cells (shHPSE) were grown in the same medium of wild-type (WT) HK-2 cells. Cells (WT and shHPSE) were grown to sub-confluence, starved in serum-free medium for 24 hours and then cultured in serum-free medium with 10, 100, 200 and 500 nM EVE for 6 hours. Fibroblast growth factor-2 (FGF-2), a growth factor that induces EMT was used as positive control. Control cultures were incubated with DMSO alone.

AKT1/2 small interfering RNA (siRNA) (sc43609, Santa Cruz Biotechnology Heidelberg, Germany) has been used to specifically silence AKT1 and AKT2 (see Additional file 1: Supplemental method and Additional file 2: Figure S1). HK2 WT cells were seeded into 6-well plates at a density of 1.5 × 10^5^ cells per well in 2 ml complete growth medium. After 24 h, the siRNA was added in serum-free medium. After 24 h the medium was replaced with fresh complete growth medium. Cells were incubated for an additional 24 h and then starved, treated with EVE (10 and 100 nM) and assayed for gene expression.

### RNA expression analysis of HPSE, α-SMA, FN, VIM and MMP-9

Total RNA was extracted from the cell monolayer using the GenElute Mammalian Total RNA Miniprep kit (Sigma-Aldrich) including DNase treatment (DNase70; Sigma). Yield and purity were assessed using Nanodrop (EuroClone) and Agilent 2100 Bioanalyzer, respectively. Total RNA from each sample was reverse-transcribed into cDNA using SuperScript II reverse transcriptase (Invitrogen). Real-time PCR were performed on an ABI-Prism 7500 using Power SYBR Green Master Mix 2 (Applied Biosystems). A quantitative analysis was performed to evaluate the expression of HPSE, MMP-9, α-SMA, VIM, FN, TGFβ2 and EGFR normalized to GAPDH. The comparative Ct method (ΔΔCt) was used to quantify gene expression, and the relative quantification was calculated as 2^-ΔΔCt^. Melting curve analysis was performed to check for any presence of non-specific amplification products.

### Immunofluorescence for α-SMA, VIM and FN

WT and HPSE-silenced cells were seeded in 22-mm glass dishes and cultured to subconfluence, serum-starved for 24 h, and then incubated with or without EVE for 24 h to analyze α-SMA, VIM and FN protein expression. Cells were fixed in 4% paraformaldehyde and permeabilized in phosphate-buffered saline (PBS) + 0.2% Triton-×100. Cells were incubated with primary antibodies for α-SMA (mouse anti-α-smooth muscle actin, 1A4, Sigma), VIM (Monoclonal Mouse Anti-Vimentin, Clone V9, Dako) and FN (Anti-Fibronectin antibody [IST-9] ab6328, abcam) overnight at 4°C in PBS with 1% BSA, then washed three times for 5 min with PBS before incubating them for 1 h at 37°C with the secondary antibody (goat anti-mouse IgG-FITC; Millipore) in PBS with 1% BSA. Nuclei were counterstained with Hoechst 33258.

### Zymography for MMP9

Gelatin substrate zymography was used to assess MMP9 activity in WT and shHPSE HK-2 cell conditioned media. Conditioned media were prepared by incubating sub-confluent cells in serum-free medium for 24 h, then with EVE at different dosages for a further 24 h. Equal amounts of conditioned media were resolved in non reducing sample buffer on 10% SDS-polyacrylamide gels co-polymerized with 0.1% gelatin. After electrophoresis, the gels were washed twice for 30 min in 2.5% Triton X-100 at room temperature to remove SDS, then equilibrated for 30 min in collagenase buffer and finally incubated overnight with fresh collagenase buffer at 37°C. After incubation, gels were stained in 0.1% Coomassie Brilliant Blue R-250, 30% MetOH/10% acetic acid for 1 h and destained in 30% MetOH/10% acetic acid. Digestion bands were analyzed using ImageJ software.

### Migration assay

Briefly, a denuded area was generated on a quiescent cell monolayer of HK-2 cells by scratching with a sterile pipette tip. The monolayer was washed twice with PBS and then incubated with medium containing the drug. Each experimental condition was tested in triplicates. The cells were photographed at different time points. The scratch area was measured in each photo to obtain a mean value. Migration was reported as the difference (in mm^2^) between the scratch dimensions observed at the baseline and after 24 hours.

### Microarray analysis

For microarray analysis we used only cells treated with 100 nM EVE because it was the lowest concentration able to trigger EMT phenotypic changes in our HK2 cells. Then, the labeled complementary RNA (cRNA) was produced using the Low Input Quick Amp Labeling (LIQA) kit (Agilent Technologies), and hybridized for 17 hours at 65°C on the Agilent SurePrint G3 Human GE 8x60K Microarray slide (Agilent Technologies). In particular it comprises more than 41,000 features, representing 34,127 human Entrez Gene RNAs. After hybridization the slides were washed according to Agilent protocols and finally scanned using the High-Resolution Microarray C Scanner (Agilent Technologies). The image files obtained by this procedure were processed using the Agilent Feature Extraction software (v10.7.3).

### Statistical analysis

Mean ± S.D. of the real-time PCR data were calculated with Rest2009 software. Differences between WT and HPSE-silenced cells, or between pre- and post-EVE treatment, were compared using Two-tailed Student’s t-test. A p value < 0.05 was set as the level of significance for all tests.

For microarray analysis, we selected, according to Groger CJ et al. [34], a total of 115 gene probe sets (corresponding to 83 genes) involved in EMT. The preprocessed microarray data were imported into the R language for statistical analysis computing (http://www.r-project.org). Genes displaying differential expression between pre and post-EVE treatment were detected using a t-test. Gene probe sets were sorted after significant p-value and were adjusted to account for multiple testing using the FDR method of Storey and Tibshirani [35].

## Results

### Everolimus-induced matrix metalloproteinase 9 (MMP9) gene expression

**Figure 1:**
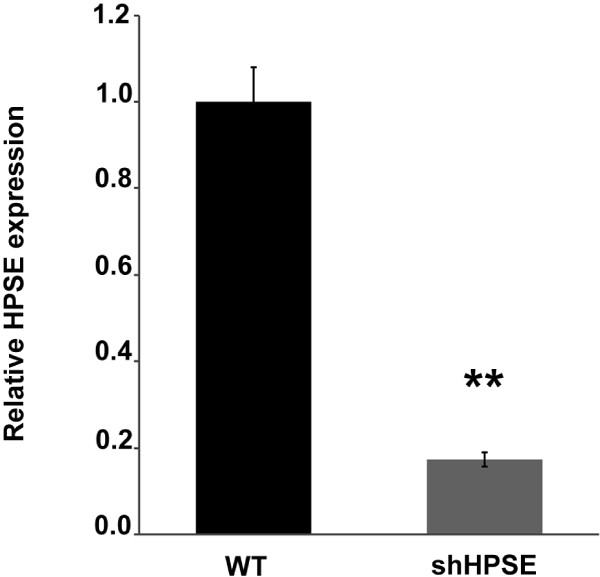
**Heparanase (HPSE) silencing efficiency evaluated by RT-PCR.** Histogram represents the relative HPSE gene expression in wild-type (WT) and HPSE-silenced (shHPSE) cell lines. The very low HPSE level in shHPSE demonstrates the good efficiency of cellular silencing procedure. **p < 0.001 versus WT.

To evaluate whether EVE treatment was able to modulate MMP9 transcription in wild-type (WT) and HPSE-silenced HK-2 cells (shHPSE) [Figure 1], we first treated for 6 hours both cell lines with EVE (10, 100, 200 and 500 nM) and FGF-2 (10 ng/ml), a growth factor involved in EMT and, then, we measured MMP9 gene expression by real-time PCR.

**Figure 2:**
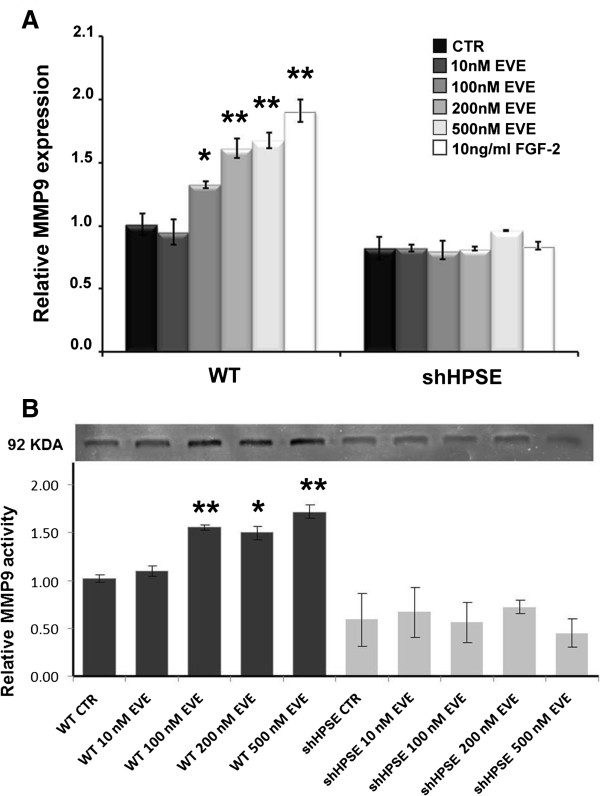
**Matrix metalloproteinase-9 (MMP9) gene expression and activity after Everolimus (EVE) treatment. (A)** Histograms represent MMP9 mRNA expression levels determined by real-time PCR in WT and HPSE silenced HK-2 cells (shHPSE). WT and silenced cell lines were cultured with 10, 100, 200 and 500 nM Everolimus for 6 h. FGF-2 (10 ng/ml), a well-known EMT inducer, was used as positive control. Mean ± S.D. (error bars) of three separate experiments performed in triplicate. *p < 0.05 vs WT control cells (CTR); **p < 0.001 versus WT CTR. **(B)***Upper*, gelatin zymography shows MMP-9 activity bands in the serum-free conditioned media of WT and silenced HK2 cells. WT and silenced cell lines were cultured with 10, 100, 200 and 500 nM Everolimus for 24 h, then conditioned media were harvested. *Lower*, histogram represents the densitometric analysis of relative MMP-9 activity. Mean ± S.D. (error bars) of three separate experiments performed in triplicate. *p < 0.05 vs WT control cells (CTR); **p < 0.001 versus WT CTR.

As showed in Figure 2A, only high EVE dosages (100, 200 and 500 nM) significantly increased the MMP9 expression level, while 10 nM EVE (corresponding to therapeutic concentration) did not induce any modulation of this EMT marker.

Otherwise, in shHPSE cells, EVE did not induce any change in the expression level of this proteinase.

### MMP9 Activity after everolimus treatment

To assess if the MMP9 protein level mirrors the increased mRNA expression, we measured the extracellular MMP9 activity by gelatin zymography on conditioned media of WT and shHPSE cells. Our data showed, similarly to RT-PCR, that only high EVE dosages (100, 200 and 500 nM) significantly triggered the release of active MMP9 by WT tubular cells, whereas this drug had no effect on HPSE-Silenced cells [Figure 2B]. No effects were observed in both cell lines after incubation with 10 nM EVE.

### Alpha-SMA (αSMA), vimentin (VIM) and fibronectin (FN) gene expression

Subsequently, to better define EVE-induced EMT, we measured the expression level of other three well known EMT markers: αSMA, VIM and FN.

High concentrations of EVE (200, 500 nM), similarly to FGF-2 (10 ng/ml), increased α-SMA, VIM and FN expression level in WT tubular cells. One-hundred nM EVE induced a significant α-SMA and FN up-regulation, but it was unable to determine a change in the VIM expression level. Similarly to MMP9, we did not observe any EVE-induced gene expression modulation of these markers in HPSE shRNA cells.

**Figure 3:**
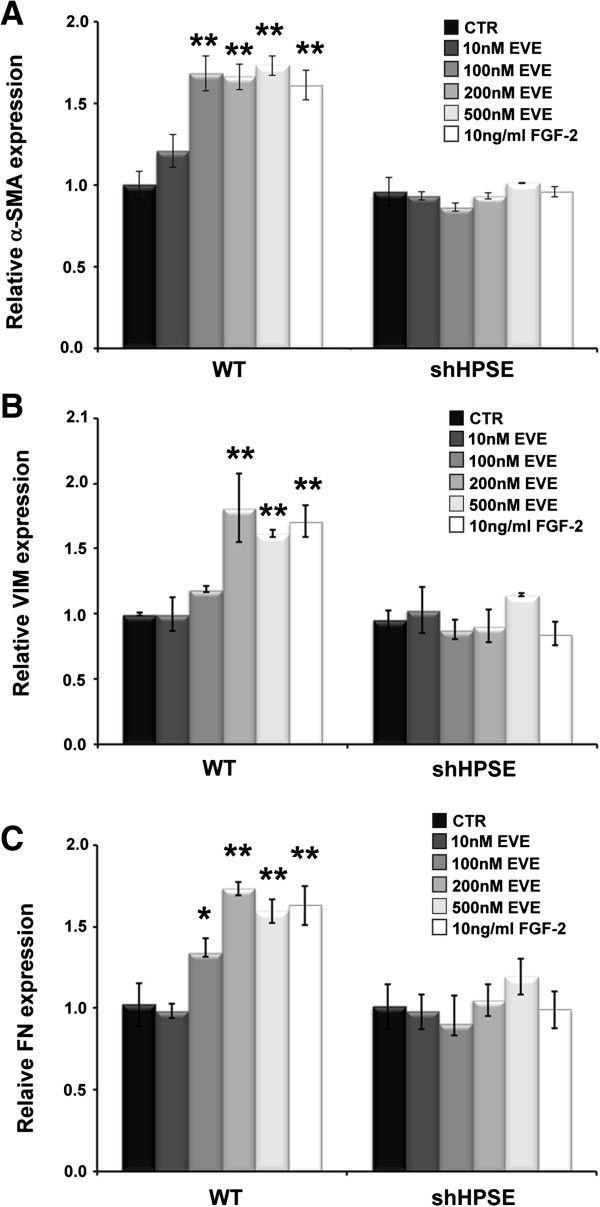
**Alpha-SMA (α-SMA) Vimentin (VIM) and Fibronectin (FN) gene expression after Everolimus (EVE).** Histograms represent **A)** α-SMA, **B)** VIM and **C)** FN mRNA levels determined by real-time PCR. WT and silenced cell lines were cultured with Everolimus (10, 100, 200 and 500 nM) for 6 h. FGF-2 (10 ng/ml) was used as positive control. Mean ± S.D. (error bars) of three separate experiments performed in triplicate. *p < 0.05 vs WT control cells (CTR); **p < 0.001 versus WT CTR.

Moreover, 10 nM EVE did not induce any change in αSMA, VIM and FN expression levels [Figure 3].

### Immunofluorescence (IF) analysis

**Figure 4:**
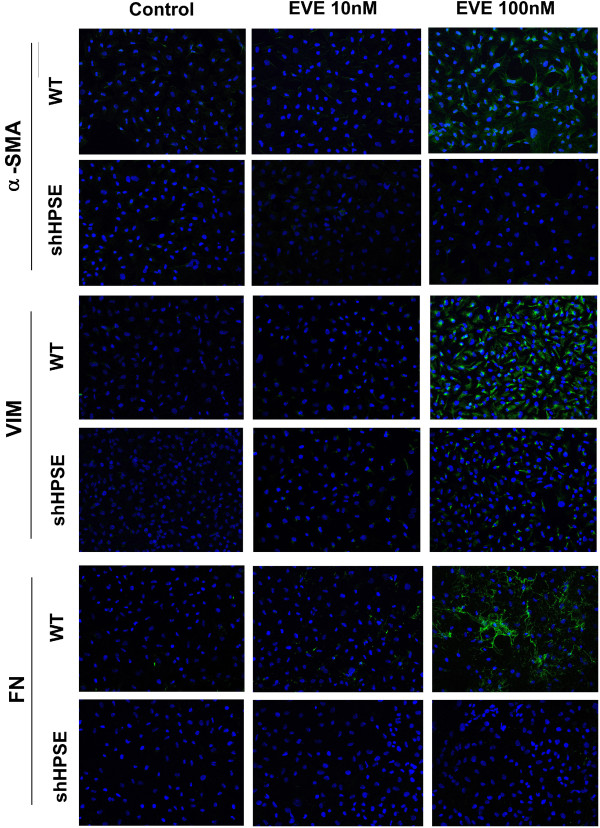
**Alpha-SMA (α-SMA), Vimentin (VIM) and Fibronectin (FN) protein expression after Everolimus (EVE).** Representative images of one of two immunostaining experiments for α-SMA, VIM and FN in WT and HPSE-silenced cells, with or without EVE treatment (magnification, ×400).

Conformingly to RT-PCR experiments, IF analysis showed that high concentration of EVE (100 nM) increased protein expression of α-SMA, VIM and FN in WT HK2 cells. No effects were seen in HPSE-silenced cells. Additionally, cells treated with 10 nM EVE did not show any change in the protein expression of the above mentioned mesenchymal markers [Figure 4].

### Cell motility

**Figure 5:**
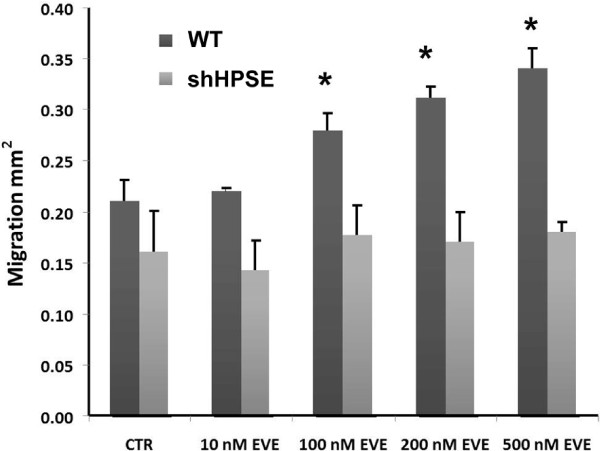
**Cell migration induced by Everolimus (EVE).** Histogram represents migration (in mm^2^) over 24 h in WT and silenced cell lines after treatment with Everolimus (10, 100, 200 and 500 nM). Data are presented as mean ± S.D. (error bars) of three separate experiments. *p < 0.05 vs WT control cells (CTR).

During EMT, renal tubular epithelial cells acquire the ability to migrate through the basal membrane into the interstitium. We showed that only high EVE doses were able to induce significant cell motility in WT cells. HPSE silenced cells did not show this property. EVE 10 nM was unable to determine also this biological effect [Figure 5].

This result suggests that the therapeutic dosage of EVE does not induce EMT.

### Role of AKT

Since mTORC1 inhibition may lead to AKT activation and since AKT pathway has a central role in EMT, we investigated the effect of EVE in AKT-silenced cells.

**Figure 6:**
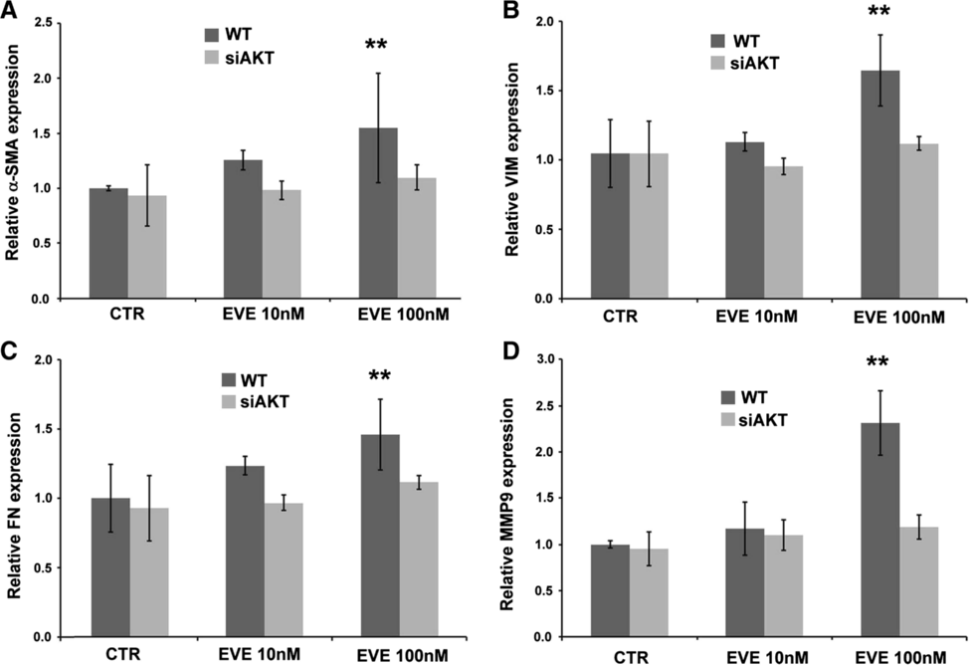
**α-SMA, VIM, FN and MMP9 expression in WT and AKT-silenced cells treated with EVE.** Histograms represent **A)** α-SMA, **B)** VIM, **C)** FN and **D)** MMP9 mRNA expression levels determined by real-time PCR. WT and AKT-silenced cells were cultured with Everolimus (10 and 100 nM) for 6 h. Mean ± S.D. (error bars) of three separate experiments performed in triplicate. **p < 0.001 versus WT CTR.

Silencing of AKT did not modify α-SMA, VIM, FN and MMP9 basal expression levels but prevented their increase in response to 100 nM EVE [Figure 6].

### Microarray

**Figure 7:**
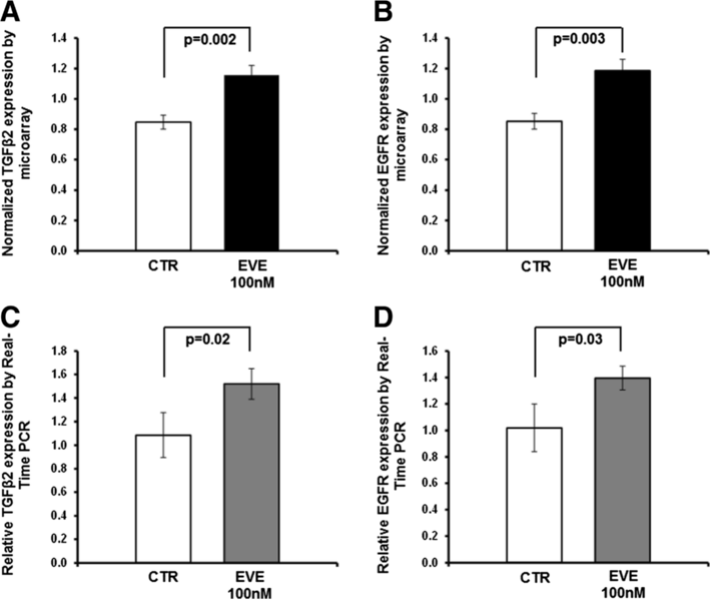
**Up-regulated genes in HK2 cells after 100 nM EVE treatment.** Histogram represents the normalized expression level of TGFβ2 **(A)** and EGFR **(B)** in un-treated (CTR) and EVE-treated HK2 cells (EVE 100 nM) determined by microarray. P value performed by two-sample t-test. Histograms **C** and **D** represent, respectively, TGFβ2 and EGFR mRNA expression levels determined by real-time PCR. WT cells were cultured with 100 nM Everolimus for 6 h. Mean ± S.D. (error bars) of three separate experiments performed in triplicate. P value was calculated by two-sample t-test.

In order to confirm results obtained by classical bio-molecular techniques and to find new biological elements involved in EVE-induced EMT, we analyzed the differences in expression of 83 EMT-related genes in HK-2 cells between pre- and post-EVE treatment. Interestingly, after statistical analysis, we identified other 2 genes significantly up-regulated in EVE-treated cells (p < 0.005 and FDR < 5%): transforming growth factor beta 2 (TGFβ2) and epidermal growth factor receptor (EGFR) [Figure 7A and B]. Gene expression analysis by real-time PCR confirmed the aforementioned results [Figure 7C and D]. Additionally, α-SMA, VIM, FN and MMP9 mRNA levels were higher in EVE-treated cells compared to CTR confirming our previous results (see Additional file 3: Figure S2).

## Discussion

Since the introduction in renal transplant therapy, mTOR inhibitors (mTOR-I, Sirolimus and Everolimus) have been considered promising immunosuppressant due to their relatively low nephrotoxicity [36,37]. The main mechanism of action of these drugs is the inhibition of cell signaling through the PI3K/Akt/mTOR pathway [38-40].

mTOR is a large protein belonging to the phosphoinositide kinase-related kinase family. The carboxy-terminal portion of mTOR contains both the kinase and the FKBP-rapamycin binding (FRB) domain. In mammals, mTOR associates with mammalian lethal with SEC13 protein 8 (mLST8), proline-rich AKT substrate of 40 kDa (AKT1S1) and regulatory-associated protein of mTOR (RAPTOR) to form the rapamycin-sensitive mTOR complex 1 (mTORC1). The mTORC1 activates protein synthesis through modulation of the 40S ribosomal protein S6 kinase (S6K) and the translational initiation factor eIF-4E binding protein 1 (4E-BP1). mTORC1 is acutely sensitive to inhibition by Sirolimus/Everolimus. Both drugs interact in mammalian cells with the immunophilin FKBP12, and the FKBP12–rapamycin complex then binds to the FRB domain in mTOR. On docking to the FRB domain, which is in close proximity to the catalytic site, the FKBP12–rapamycin complex allosterically inhibits mTORC1 kinase activity by an unknown mechanism [41]. These biological effects confer to these drugs important immunosuppressive and anti-proliferative properties.

Despite this potential, numerous published reports have described important EVE-related adverse effects in organ transplant recipients (e.g., impaired wound healing, increased risk of dyslipidemia, proteinuria and prolonged recovery of delayed graft function) [11-13].

Particularly, in the last years, there have been described several interstitial pulmonary fibrosis events following mTOR-I administration [13-17]. Although, the ethiopathogenetic mechanism associated to these pulmonary diseases is still not completely defined, the activation of a partial EMT in bronchial epithelial cells treated with mTOR-I seems to have a pivotal role [18-20]. These cells, in fact, showed higher protein expression of mesenchymal markers including fibronectin and vimentin [19].

Therefore, to evaluate whether EVE treatment was able to induce EMT in human proximal tubular (HK-2) cells, we measured, by RT-PCR, changes in expression level of four genes encoding for well known EMT markers (MMP-9, α-SMA, Fibronectin and Vimentin) in wild-type (WT) and HPSE-silenced HK2 cells incubated for 6 hours with 10, 100, 200 and 500 nM EVE. We chose to test in vitro high EVE concentrations (more than 100 nM), because corresponding to dosage frequently used in chemotherapeutic protocols.

Our results demonstrated that WT HK-2 cells cultured with high concentrations of EVE (from 100 to 500 nM) exhibited an up-regulation of all four EMT markers both at gene and protein level. Additionally, these dosages induced the increase of MMP-9 enzymatic activity and a significant cellular migration in the same cell lines.

On the other side, low dose of EVE (10 nM), usually used for organ transplantation, was unable to induce EMT in WT cells. This is in line with several published papers reporting potential anti-fibrotic kidney properties of both mTOR-I [42,43]. Pontrelli et al. have recently reported that rapamycin, reducing Plasminogen activator inhibitor-1 (PAI)-1 expression, was able to decrease extracellular matrix (ECM) deposition in all renal compartments of patients with chronic allograft nephropathy [44].

**Figure 8:**
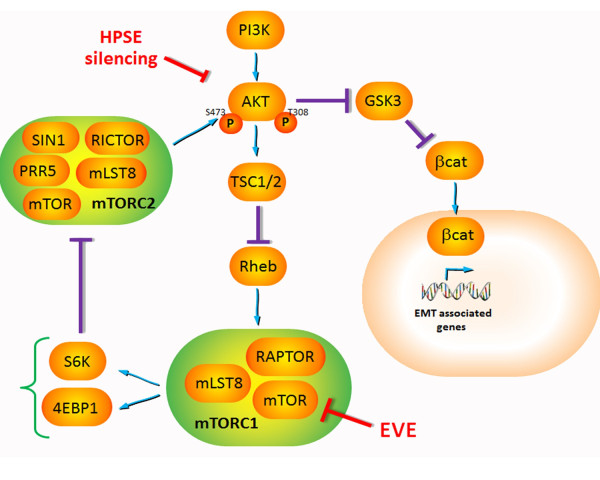
**Supposed mechanism of EVE-induced EMT.** The treatment with high doses of Everolimus activates the EMT pathway in tubular cells. It is known that mTORC2 complex participates in AKT activation (S473 phosphorylation) and AKT is a central signal pathway in renal EMT. Therefore, high concentrations of EVE, through a massive mTORC1 inhibition, may lead to a down-regulation of S6K and a subsequent hyper-activation of mTORC2 that, sustaining the phosphorylation of AKT at S473, could induce a feedback loop that stimulates PI3K-AKT signaling activating the cellular/molecular machinery leading to renal fibrosis. In particular AKT, once activated, could induce, through the inhibition of GSK3, the nuclear translocation of β-catenin which stimulates the expression of EMT-associated genes.

On the other hand, high concentrations of EVE, through a massive mTORC1 inhibition, may lead to a down-regulation of S6K and a subsequent hyper-activation of mTORC2 that, sustaining the phosphorylation of AKT at S473, could induce a feedback loop that stimulates PI3K-AKT signaling activating the cellular/molecular machinery leading to renal fibrosis [45-48]. In particular AKT, once activated, could induce, through the inhibition of Glycogen synthase kinase 3 (GSK3), the nuclear translocation of β-catenin which stimulates the expression of EMT-associated genes [49] [Figure 8]. Our data confirmed that the knock-down of AKT can control the activation of EMT program.

This confirms previous results from pharmacodynamic analysis of cancer patient–derived tumor material showed increased AKT S473 phosphorylation in some cases [50,51] after treatment with doses and schedules of EVE defined as biologically optimal through pharmacokinetic/pharmacodynamic modeling of preclinical and phase I data [52-54].

Additionally, we emphasized that, as previously demonstrated, HPSE has a pivotal role in the aforementioned pathway [27,28]. In fact, the silencing of this enzyme in our cellular model reversed the activation of the EMT [55,56].

Heparanase (HPSE) is an endo-β-D-glucuronidase that cleaves heparan sulfate (HS) side chains at a limited number of sites, hence participates in ECM degradation and remodeling [57,58]. The degradation of several constituents of the ECM, including heparan sulfate proteoglycans (HSPG), promotes the release of growth factors such as FGF-2. Moreover, we previously shown that HPSE is necessary to sustain the PI3K/AKT pathway mediated by FGF-2 which induces the expression of mesenchymal markers α-SMA and Vimentin (VIM), leads to degradation of the basement membrane by means of the secretion of matrix metalloproteinases (MMPs) and increases cell motility [26]. The heparanase expression is finely regulated by transcription factor, DNA methylation and various endogenous molecules [59-61].

Finally in order to find new elements involved in EVE-induced EMT, we analyzed the differences in the transcriptomic profile (83 EMT-related genes) between HK-2 EVE-treated cells and controls. Our study was performed using a microarray technology able to evaluate simultaneously the expression of more than 30,000 genes. However, to take full advantage of the opportunities offered by this high throughput method, it is necessary to manage, integrate and interpret a huge amount of data correctly. Thus, we decided to use a pathway analysis to focus our research on candidate genes known to be associated with EMT in order to reduce the false positive rate and the puzzling factors not directly associated with the aims of our research.

Different statistical algorithms identified two genes (*TGFβ2* and *EGFR*) significantly up-regulated by this drug.

Transforming growth factor-beta 2 (TGF-β2) is a secreted signaling molecule that regulates a diverse range of cellular responses, including proliferation, differentiation, migration and apoptosis [62]. Even if the TGF-β1 isoform has been largely characterized as EMT trigger in kidney, also TGF-β2 is a well defined key mediator of EMT-induced fibrosis in both experimental and human kidney diseases [63-66].

Epidermal growth factor receptor (EGFR) is a transmembrane protein receptor with tyrosine kinase activity that triggers numerous signaling pathways involved in diverse cell functions (e.g., proliferation, cell survival) and it has been recently considered a key role of EGFR in TGFβ-dependent tubulointerstitial EMT-induced fibrosis [67].

Interestingly, although renal EMT-related effects were reached in our model only with very high concentration of this drug, we can not exclude that other different cells (including pneumocytes, bronchial epithelium cells) or patients with a genetic predisposition could present this condition after exposure to lower or therapeutic dose of EVE. This assumption is in line with a recent work published by Xu X et al. describing a pro-fibrotic effect of mTOR inhibitors in lung epithelial cells [68]. However, our hypothesis, although suggestive, need to be better addressed and validated in future *in vivo* studies.

Finally, our results, if confirmed by additional studies, could be useful for researchers to develop new therapeutic strategy that may prevent/minimize the systemic fibrotic adverse effects induced by EVE therapy.

Altogether, our data, although obtained by an *in vitro* model, reveal new biological/cellular aspects of the renal and systemic pro-fibrotic machinery induced by EVE treatment.

## Conclusions

Our *in vitro* study reveals new biological/cellular aspects of the pro-fibrotic activity of EVE and it demonstrates, for the first time, that an heparanase-mediated EMT in renal tubular cells may be activated by high doses of this drug. Additionally, our results, confirming several literature evidences [69], suggest that clinicians should administer the adequate dosage of EVE in order to increase efficacy and reduce adverse effects. Finally HPSE could be a new potential therapeutic target useful to prevent/minimize mTOR-I-related systemic fibrotic adverse effects.

## Competing interests

The authors declare that they have no competing interests.

## Authors’ contributions

VM, SG and MO performed biomolecular experiments and wrote the paper. GZ carried out microarray and statistical analysis, contributed to the design of the study and wrote the paper. GG and AL helped in the manuscript writing and data analysis. All authors read and approved the final manuscript.

## Supplementary Material

Additional file 1 **Supplemental method.** Measurement of AKT protein level by western blot in WT and AKT-silenced HK2 cells before and after Everolimus treatment (10 and 100 nM). Click here for file

Additional file 2: Figure S1 Total AKT protein level in WT and AKT1/2-silenced HK2 cells. As showed, measurement of total AKT protein levels by western blotting confirmed the AKT1/2-silencing of HK2 cells in each experimental points utilized for EMT gene expression analysis (see Figure 6 included in the main manuscript). GAPDH was included as the loading control. Bottom: quantitative analysis of three experiments. p value^1^ calculated by ANOVA: CTR versus EVE 10 nM versus EVE 100 nM. p value^2^ calculated by ANOVA: siAKT versus siAKT EVE 10 nM versus siAKT 100 nM. Click here for file

Additional file 3: Figure S2 α-SMA, VIM, FN, and MMP9 gene expression after Everolimus (EVE) treatment. Histogram represents the normalized expression level by microarray of α-SMA (A), VIM (B), FN (C) and MMP9 (D) in un-treated (CTR) and EVE-treated HK2 cells (100 nM for 6 h). Mean ± SD of three separate experiments performed in triplicate. P value performed by two-sample t-test. Click here for file
