# Supplementary material for: Everolimus-induced epithelial to mesenchymal transition in immortalized human renal proximal tubular epithelial cells: key role of heparanase
Source: J Transl Med. 2013 Nov 20;11:292. doi: 10.1186/1479-5876-11-292 (PMC4222256; doi:10.1186/1479-5876-11-292)
Supplement: Additional file 1 — Supplemental method. Measurement of AKT protein level by western blot in WT and AKT-silenced HK2 cells before and after Everolimus treatment (10 and 100 nM). [file 1479-5876-11-292-S1.doc]

**Supplement Information for:**

**EVEROLIMUS-INDUCED EPITHELIAL TO MESENCHYMAL TRANSITION IN IMMORTALIZED HUMAN RENAL PROXIMAL TUBULAR EPITHELIAL CELLS: KEY ROLE OF HEPARANASE**

Valentina Masola^1*^, Gianluigi Zaza^1*^, Simona Granata^1^, Giovanni Gambaro^2^, Maurizio Onisto^3^, Antonio Lupo^1^

^1^ Renal Unit, Department of Medicine, University-Hospital of Verona, Italy

^2^ Division of Nephrology and Dialysis, Columbus-Gemelli Hospital Catholic University, School of Medicine Rome, Italy

^3^ Department of Biomedical sciences, University of Padova, Italy

* Valentina Masola and Gianluigi Zaza equally contributed to the present study.

**Address correspondence to:**

Gianluigi Zaza, MD, PhD

Renal Unit, Department of Medicine,

University-Hospital of Verona, Verona, Italy.

Piazzale A. Stefani 1, 37126, Verona (VR), Italy

Tel. 045.8122528;

fax 045.8027311

E mail: [gianluigi.zaza@univr.it](mailto:gianluigi.zaza@univr.it)

Table of Contents

**1. Supplemental Methods**

1.1. Western blot for AKT

1.2. α-SMA, VIM, FN and MMP9 gene-expression analysis by Microarray technology

**2. Supplemental Results**

2.1. AKT silencing performance evaluated by protein analysis

2.2. Gene expression level of α-SMA, VIM, FN and MMP9 discriminated HK-2 between pre- and post-EVE-treatment.

Figure S1: Western blot analysis of total AKT protein level in WT and AKT1/2-silenced HK2 cells treated or not with EVE.

Figure S2: α-SMA, VIM, FN and MMP9 gene expression after Everolimus (EVE) treatment.

**1. Supplemental Methods**

**1.1. Western blot for AKT**

AKT-silenced HK2 cells were sonicated, centrifuged at 4°C for 30 min at 15,000g and the total proteins in the supernatant were quantified using the Bio-Rad Protein Assay. Equal amount of proteins from each sample (50 μg) were treated in reducing sample buffer (2% SDS, 80 mM Tris–HCl [pH 6.8], 10% glycerol, 0.005% bromophenol blue, 10% β-mercaptoethanol) and denatured 10 min at 100°C. Then samples were separated by 10% SDS–PAGE (120 V–25 mA) and electro-transferred to nitrocellulose membranes (300 V–350 mA) for 2.5 h at 4°C. The membranes were blocked with fat-free milk (5%) in TBST buffer (20 mM Tris–HCl [pH 7.4], 150 mM NaCl, 0.05% Tween-20) for 1 h at room temperature. Membranes were incubated with primary polyclonal antibody anti-AKT (GTX121937, GeneTex) and anti-GAPDH (sc-25778 Santa Cruz Biotechnology) overnight at 4°C and after washing in TBST buffer, they were incubated with a secondary peroxidase-conjugated antibody at room temperature for 1 h. The signal was detected using the “SuperSignals West Pico Chemiluminescent” substrate solution (Pierce, Rockford, IL, USA), according to the manufacturer's instructions.

**1.2. α-SMA, VIM, FN and MMP9 gene-expression analysis by Microarray technology**

For microarray analysis we used only cells treated with 100 nM EVE because it was the lower concentration able to trigger EMT phenotypic changes in our HK2 cells. Then, the labeled cRNA was produced using the Low Input Quick Amp Labeling (LIQA) kit (Agilent Technologies), and hybridized for 17 hours at 65°C on the Agilent SurePrint G3 Human GE 8x60K Microarray slide (Agilent Technologies). In particular it comprises more than 41,000 features, representing 34,127 human Entrez Gene RNAs. After hybridization the slides were washed according to Agilent protocols and finally scanned using the High-Resolution Microarray C Scanner (Agilent Technologies). The image files obtained by this procedure were processed using the Agilent Feature Extraction software (v10.7.3).

To measure differences in expression levels of four selected genes encoding for EMT markers [(α-SMA, VIM, FN and MMP9)] between untreated versus treated HK2 cell, we used a t-test. The preprocessed microarray data were imported into the R language for statistical analysis computing ([http://www.r-project.org](http://www.r-project.org/)).

**2. Supplemental Results**

**2.1. AKT silencing performance evaluated by protein analysis**

As showed in Figure S1, measurement of total AKT protein levels by western blotting confirmed the AKT1/2-silencing of HK2 cells in each experimental points utilized for EMT gene expression analysis (see Figure 6 included in the main manuscript).


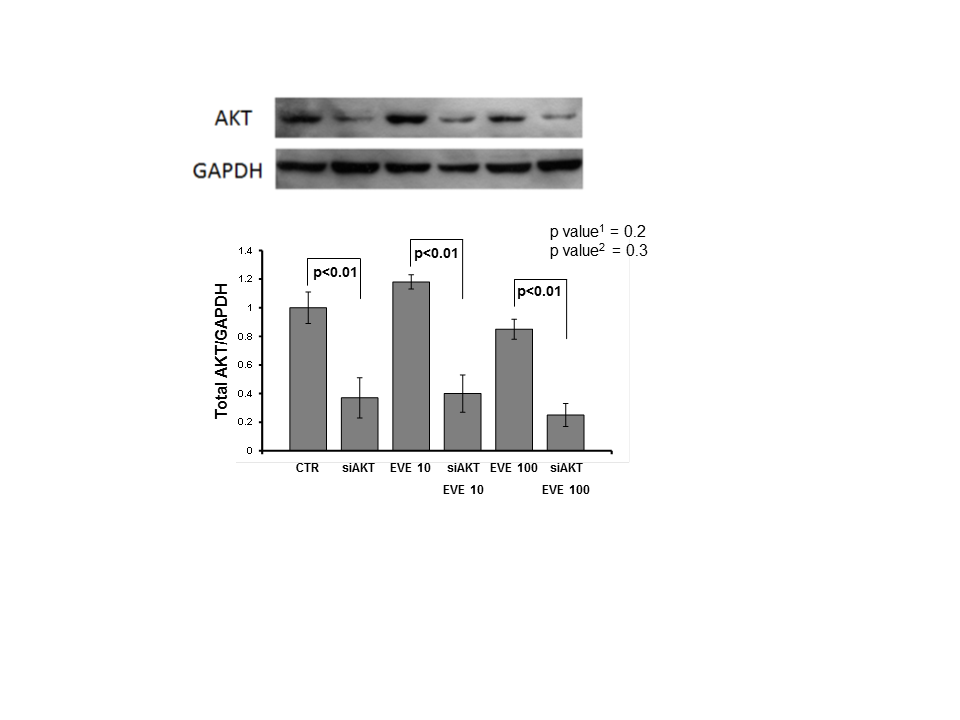


**FIGURE S1:** **FIGURE S1:** Total AKT protein level in WT and AKT1/2-silenced HK2 cells. GAPDH was included as the loading control. Bottom: quantitative analysis of three experiments.

p value^1^ calculated by ANOVA: CTR *versus* EVE 10 nM *versus* EVE 100 nM

p value^2^ calculated by ANOVA: siAKT *versus* siAKT EVE 10 nM *versus* siAKT 100 nM

**2.2. Gene expression level of α-SMA, VIM, FN and MMP9 discriminated HK-2 between pre- and post-EVE-treatment.**

Using microarray experiments, we found that VIM, FN and MMP9 resulted up-regulated in 100 nM EVE-treated HK2. For α-SMA, we found only a trend in increment in gene expression. However, the up-regulation was non statistical significant (p.value=0.08). This could be probably due to the microarray technique or quality of chip hybridization (FIGURE S2).


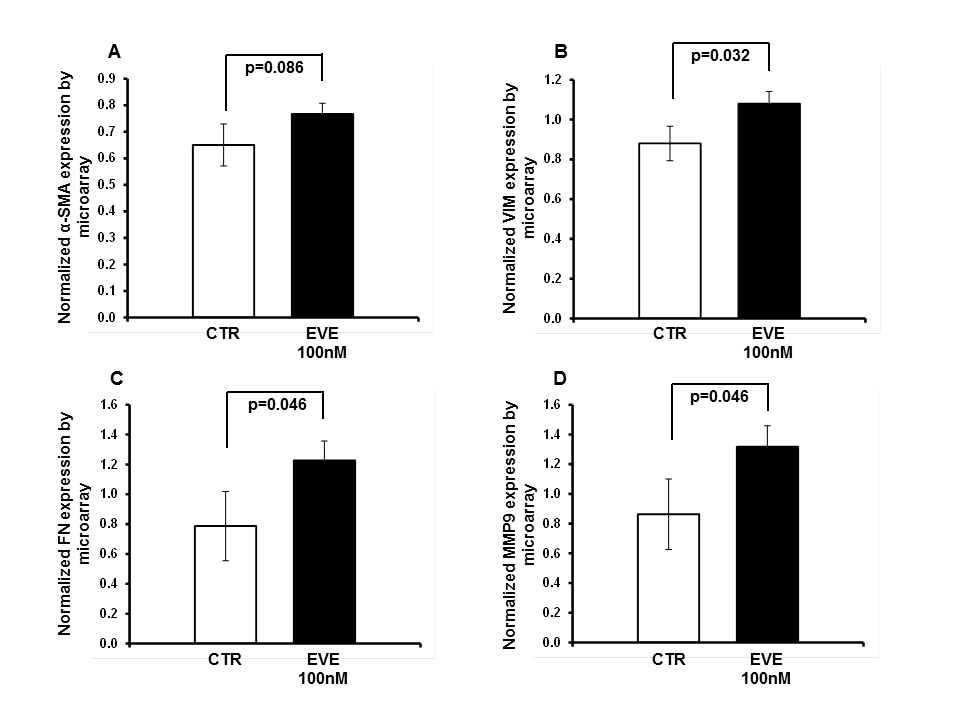


**FIGURE S2. α-SMA, VIM, FN, and MMP9 gene expression after Everolimus (EVE) treatment.** Histogram represents the normalized expression level of α-SMA (A), VIM (B), FN (C) and MMP9 (D) in un-treated (CTR) and EVE-treated HK2 cells (EVE 100nM) determined by microarray. P value performed by two-sample t-test. WT cells were cultured with 100nM Everolimus for 6 h. Mean ± S.D. (error bars) of three separate experiments performed in triplicate. P.value was calculated by two-sample t-test.
