# Supplementary figures and images for: Everolimus-induced epithelial to mesenchymal transition in immortalized human renal proximal tubular epithelial cells: key role of heparanase
Source: J Transl Med. 2013 Nov 20;11:292. doi: 10.1186/1479-5876-11-292 (PMC4222256; doi:10.1186/1479-5876-11-292)

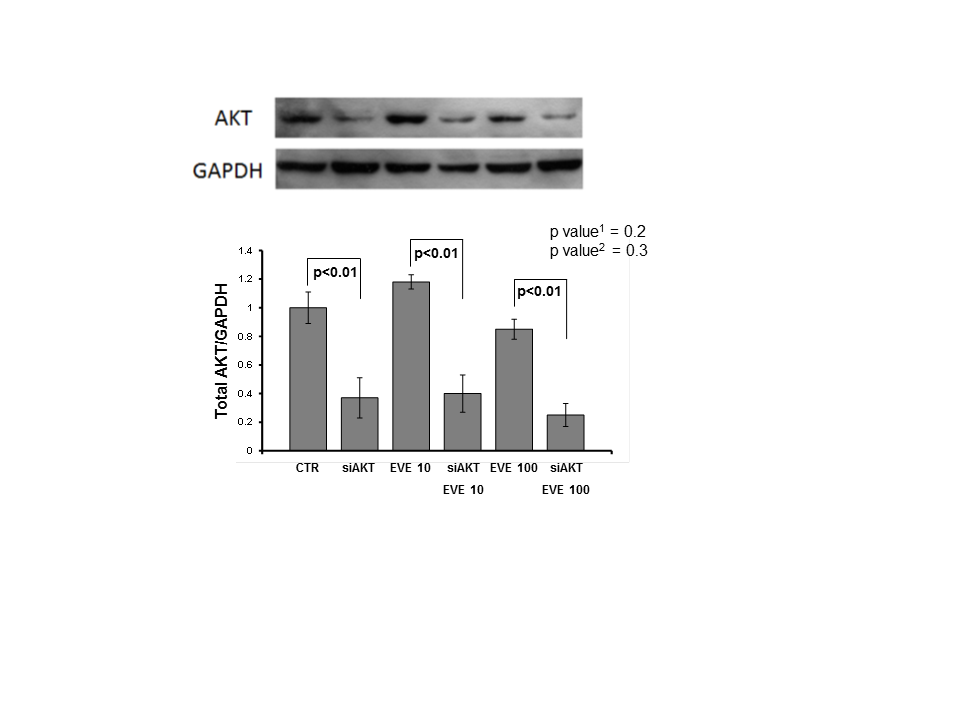

Supplement: Additional file 2: Figure S1 — Total AKT protein level in WT and AKT1/2-silenced HK2 cells. As showed, measurement of total AKT protein levels by western blotting confirmed the AKT1/2-silencing of HK2 cells in each experimental points utilized for EMT gene expression analysis (see Figure 6 included in the main manuscript). GAPDH was included as the loading control. Bottom: quantitative analysis of three experiments. p value1 calculated by ANOVA: CTR versus EVE 10 nM versus EVE 100 nM. p value2 calculated by ANOVA: siAKT versus siAKT EVE 10 nM versus siAKT 100 nM. [file 1479-5876-11-292-S2.tif]

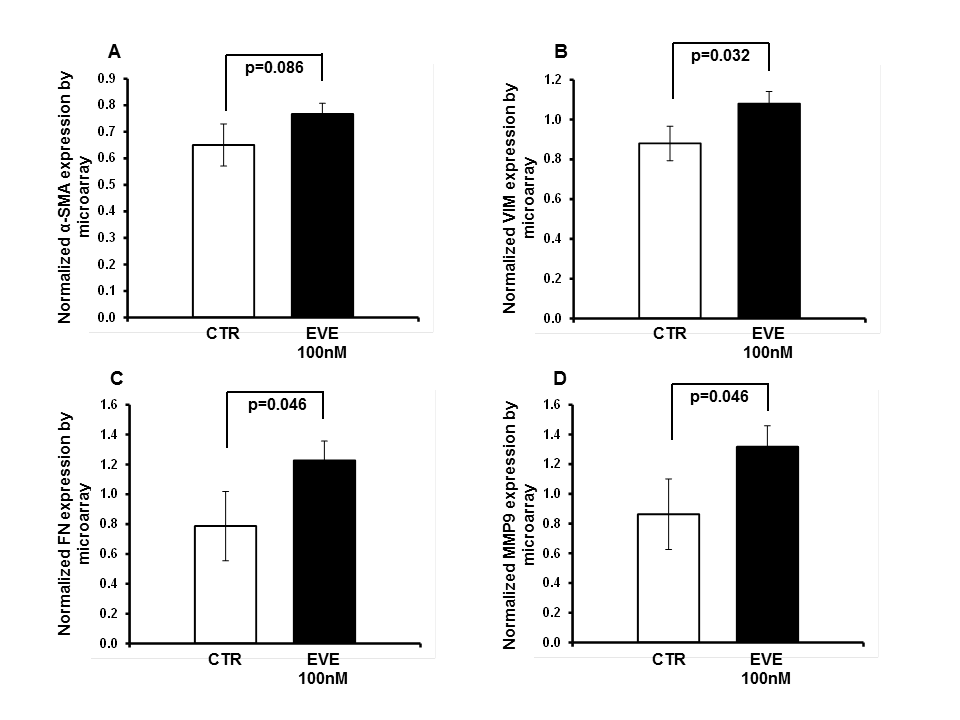

Supplement: Additional file 3: Figure S2 — α-SMA, VIM, FN, and MMP9 gene expression after Everolimus (EVE) treatment. Histogram represents the normalized expression level by microarray of α-SMA (A), VIM (B), FN (C) and MMP9 (D) in un-treated (CTR) and EVE-treated HK2 cells (100 nM for 6 h). Mean ± SD of three separate experiments performed in triplicate. P value performed by two-sample t-test. [file 1479-5876-11-292-S3.tif]
